# Supplementary material for: ‘140R’ Rootstock Regulates Resveratrol Content in ‘Cabernet Sauvignon’ Grapevine Leaves Through miRNA
Source: Plants (Basel). 2024 Oct 31;13(21):3057. doi: 10.3390/plants13213057 (PMC11548312; doi:10.3390/plants13213057)

Supplementary Materials:

Table S1. Quality summary of sequencing data

Samples	Raw_reads	Clean_reads	GC(%)	Q20(%)	Q30(%)	Mapped_Reads
CS/140R-1	29403611	20644054	47.14	99.17	97.22	5694861(69.60%)
CS/140R-2	38454093	27562948	46.71	99.21	97.28	6349532(66.45%)
CS/140R-3	36097891	26505408	46.77	99.17	97.18	7958354(71.40%)
140R/140R-1	35321513	20271006	49.01	99.18	97.14	4491753(75.09%)
140R/140R-2	38550304	21061639	48.54	98.97	96.54	6973469(75.07%)
140R/140R-3	34939153	22230573	48.16	99.26	97.46	7293950(74.08%)
CS/CS-1	37236227	30115240	45.15	99.13	97.08	11359652(75.59%)
CS/CS-2	40203934	32511076	45.17	99.24	97.37	12254643(75.73%)
CS/CS-3	42987910	34819452	45.16	99.21	97.26	13116897(75.70%)

Table S2. Notes on the classification of grapevine with small RNAs

type	Experimental repetition	CS/140R		140R/140R		CS/CS	
		Total	% of clean reads	Total	% of clean reads	Total	% of clean reads
	1	20644054	100.00%	20271006	100.00%	30115240	100.00%
clean reads	2	27562948	100.00%	21061639	100.00%	32511076	100.00%
	3	26505408	100.00%	22230573	100.00%	34819452	100.00%
	1	11888313	57.59%	12675448	62.53%	14441035	47.95%
rRNA	2	17235763	62.53%	11147803	52.93%	15629227	48.07%
	3	14630586	55.20%	11540896	51.91%	16742499	48.08%
	1	16054	0.08%	23124	0.11%	30469	0.10%
snoRNA	2	18763	0.07%	13922	0.08%	32812	0.10%
	3	23829	0.09%	17770	0.08%	35796	0.10%
	1	425787	2.06%	1538610	7.59%	453557	1.51%
tRNA	2	578641	2.10%	517905	2.46%	490525	1.51%
	3	532651	2.01%	720022	3.24%	525158	1.51%
	1	131085	0.63%	51926	0.26%	163137	0.54%
Repbase	2	174037	0.63%	92733	0.44%	176878	0.54%
	3	172124	0.65%	105520	0.47%	188467	0.54%
	1	8182814	39.64%	5981897	29.51%	15027042	49.90%
Unannotated	2	9555743	34.67%	9289276	44.10%	16181634	49.78%
	3	11146216	42.05%	9846364	44.30%	17327532	49.77%

Note: ribosomal RNA （rRNA）;transfer RNA（tRNA）;Small nucleolar RNA（snoRNA）.

Table S3. MiRNA family member

miRNA	CS/140R	140R/140R	CS/CS
vvi-miR156	9	8	9
vvi-miR159	3	3	3
vvi-miR160	5	5	5
vvi-miR162	1	1	1
vvi-miR164	4	5	5
vvi-miR166	8	8	8
vvi-miR167	5	5	5

vvi-miR168	1	1	1
vvi-miR169	23	23	23
vvi-miR171	6	6	9
vvi-miR172	2	3	3
vvi-miR2111	1	1	2
vvi-miR2950	2	2	2
vvi-miR319	5	5	5
vvi-miR3623	2	2	2
vvi-miR3624	2	2	2
vvi-miR3625	2	2	2
vvi-miR3627	2	1	2
vvi-miR3629	4	4	4
vvi-miR3630	2	2	2
vvi-miR3631	6	6	6
vvi-miR3632	2	2	2
vvi-miR3633	4	4	4
vvi-miR3634	2	2	2
vvi-miR3635	1	2	2
vvi-miR3636	2	2	2
vvi-miR3637	2	2	2
vvi-miR3638	2	2	2
vvi-miR3639	2	2	2
vvi-miR3640	2	2	2
vvi-miR390	1	1	1
vvi-miR393	2	2	2
vvi-miR394	3	3	3
vvi-miR395	13	13	14
vvi-miR396	4	4	4
vvi-miR397	1	1	1
vvi-miR398	3	3	3
vvi-miR399	8	8	8
vvi-miR403	6	6	6
vvi-miR408	1	1	1
vvi-miR477b	3	3	3
vvi-miR479	1	1	1
vvi-miR482	1	1	1
vvi-miR535	3	3	3
vvi-miR828	1	1	1
vvi-miR845	0	3	1

Table S4. Novel miRNAs identified in grape leaves from different anvil combinations

miRNA	mature_sequence	miRNA	mature_sequence	miRNA	mature_sequence
novel_miR_106	uucgugacucauugaucuucaauc	novel_miR_96	cagacggauccacaugauaga	novel_miR_103	aauauugucugcuuuagguccacu
novel_miR_108	uucgugacucauugaucuucaauc	novel_miR_158	acugagacuucauuuuuaaacuga	novel_miR_114	uugguagugauuauaggaagu
novel_miR_266	uccuaaaaucacugucaaacg	novel_miR_216	guuggaagucggugggggaac	novel_miR_52	uauugggaucuagaauaugcu

novel_miR_200	uggggauguaugcugaaccuuuu	novel_miR_169	acgaagacugugagaaauccucac	novel_miR_29	aaaccgaucuggaguuuaugggcc
novel_miR_62	auauuggaugauguuaacaug	novel_miR_99	augaagacugugaggauuccucac	novel_miR_7	ugugugacugauaugaaaauuauc
novel_miR_161	cuuucuaagagaugguacgaacuu	novel_miR_202	uggggaugcauguugaaccuuu	novel_miR_101	ugauggacugcacaauggugc
novel_miR_113	ccgacguuacgcuugagcagu	novel_miR_16	auucucccucaagggcuucua	novel_miR_146	uggcaacugagacuucauuuuuga
novel_miR_246	auauuggaugauguuaacaug	novel_miR_55	agugacuuuaggaagcguuuuuaa	novel_miR_77	ugacucauugaucuucaaucugaa
novel_miR_130	uugaguugguuaagagggcuu	novel_miR_210	uaaucuguauuucuuuugaugc	novel_miR_226	ugguuguggcaguacuccauc
novel_miR_230	ugcacaugacuagguaguaacccu	novel_miR_151	agugauuuuagaaaauguuuuu	novel_miR_233	uggaacucaucucuugaagagacg
novel_miR_149	uugaaucuuugucuaguagacu	novel_miR_84	guuggaagccggugggggacc	novel_miR_125	cuucaaagggcuauuuugaacaau
novel_miR_268	cgaggaacuuaucaugaauc	novel_miR_261	ugcauuugcaccugcaccuua	novel_miR_224	auauggaugaaauuggaucugc
novel_miR_128	uuggauucgcgcacaaacucg	novel_miR_265	auaccaacuguggaaaauuguaac	novel_miR_263	uucaugaucuaguacgggauu
novel_miR_231	cuaagugugacuucuuauuuugga	novel_miR_240	ucuucaucaucuaucaggua	novel_miR_176	uugguagugauuauaggaagu
novel_miR_21	gaugaucugugaaaaggcuaaagc	novel_miR_117	ugacucauugaucuucaaucugaa	novel_miR_31	ucccaggagagauggcaccugc
novel_miR_179	uaaacacuuucuagaaucacu	novel_miR_54	gugacucauugaucuucaaucuga	novel_miR_178	uguaggccuuaaggcuuaggacgu
novel_miR_119	uucuaguuuaugcaauucacu	novel_miR_64	cauuaucuuaaaacaacucua	novel_miR_118	aucuuggagcggcuauugacuacu
novel_miR_259	gacggcaacuguagcuuaugcugc	novel_miR_243	cuuucugcuauagucauucugc	novel_miR_214	aaacacuuucuagaaucacu
novel_miR_129	auuucucuuuaucauguagacaug	novel_miR_120	uuguaguuuucuugugugauc	novel_miR_122	uugguagugauuauaggaagu
novel_miR_236	uuaaaaacguuuucuagaaucacu	novel_miR_247	auuucucuuaacccuguagacgcg	novel_miR_198	uuacacagagagaugacggugg
novel_miR_207	uggggaugcauguugaaccuuu	novel_miR_109	caucgaucucgcaccucaucg	novel_miR_183	ugaagagguugagaguggaaug
novel_miR_33	uggggaugcauguugaaccuuu	novel_miR_147	auucuccggcuggaaggcaacuuc	novel_miR_144	uuaacaggagugggugguucuuucc
novel_miR_212	uguagggaguagaaugcagc	novel_miR_30	uuugagagugauuuuagaaaa	novel_miR_180	ccaucgguuugaaauuucauc
novel_miR_234	ucacuauaucaucagccuuug	novel_miR_220	agugauuuuagaaaauguuuuu	novel_miR_171	agaagagagagaguacagcua
novel_miR_126	uuaaugucuacaugugaucugu	novel_miR_87	cacguguugccucuucauugg	novel_miR_238	uuuagcugaauagaaaaaauc

novel_miR_22	gcgugucuuggcuuggcuca	novel_miR_145	aucauguugaaagguuguucuguu	novel_miR_196	cuugucgugucccauaggauugug
novel_miR_225	guuggaagucggugggggacc	novel_miR_137	augacuuguacauacucauuuaca	novel_miR_185	augugaccguuggagccuagcucc
novel_miR_267	gugacucauugaucuucaaucuga	novel_miR_90	uggcgacaugggggcgaaa	novel_miR_66	uuugagagugauuuuagaaaa
novel_miR_208	agacuacgauuuugaauuuaaacu	novel_miR_197	uaugggcccauguaauuuugagg	novel_miR_97	uuggcccuucucccucucaug
novel_miR_98	uaaacacuuucuagaaucacu	novel_miR_42	uccuaaaaucacugucaaacg	novel_miR_160	uugguagugauuauaggaagu
novel_miR_91	uucgugacucauugaucuucaauc	novel_miR_72	ugaaacgacgucguugauagugau	novel_miR_248	uugcaugacaaaagauacuuc
novel_miR_134	uaguauuuugacuaaggaaucuuc	novel_miR_60	ugagcucuaacauuggugaag	novel_miR_163	uaaagaaauuuaaagaaaaggaac
novel_miR_256	uggggauguaugcugaaccuuuu	novel_miR_67	uuuuaauuuugauauggcaaguca	novel_miR_95	ucccaggagagauggcaccugc
novel_miR_73	uugacuuugguguuuuggacc	novel_miR_172	cugggcuuuuguuucuucgug	novel_miR_27	uggggauguaugcugaaccuuuu
novel_miR_17	uuuuuaggacuguaccuuaggguac	novel_miR_89	gugauuauaggaaguguuucu	novel_miR_167	guuggaagccgguggggggacc
novel_miR_38	aaacacuuucuagaaucacu	novel_miR_105	augugaccguuggagccuagcucc	novel_miR_34	gugauuauaggaaguguuucu
novel_miR_187	uuacgauauacuuacauuggu	novel_miR_138	uccuaaaaucacugucaaacg	novel_miR_49	uccuaaaaucacugucaaacg
novel_miR_68	cgcagaaucuuugagcggcgcgug	novel_miR_215	gcuggaagccgaugggggacc	novel_miR_110	cuuucugcuaaagucaucugc
novel_miR_153	agugacuguaccuuagguac	novel_miR_23	uaaacacuuucuagaaucacu	novel_miR_209	auauuggaugauguuaacaug
novel_miR_170	ugauuugaugauaggauugua	novel_miR_255	uaugaucugagcugucgauac	novel_miR_241	uuuguuuggucugaagcucauc
novel_miR_189	cacguguugccucuucauugg	novel_miR_177	aaacacuuucuagaaucacu	novel_miR_250	cucaugaucuaguuguagcucaaa
novel_miR_242	uuugagagugauuuuagaaaa	novel_miR_154	ucgcgugucuuggcuuggcuc	novel_miR_143	ugccaagaagcacauuccucc
novel_miR_190	cagguguagcaucaucaagauu	novel_miR_244	agugauuuuagaaaauguuuuu	novel_miR_28	uugaucggaugccaaagcuuc
novel_miR_76	cguuugguagugauuauagga	novel_miR_222	cagugauuuuagaaaauguuucua	novel_miR_182	aaguggcugccaugugaagugggc
novel_miR_252	gaugaggaucuuuggaugcaa	novel_miR_36	gugauuauaggaaguguuucu	novel_miR_35	caaagaccgaucucuuguguaagg
novel_miR_127	guuggaagccggugggggacc	novel_miR_245	ugggcuggcagcaccacc	novel_miR_121	ugauuauaggaaguguuucu
novel_miR_20	uaaaaucacugucaaacggacucu	novel_miR_133	uuugcugcuccucugguacuguuu	novel_miR_57	uuucuuguaguaaaucaugca

novel_miR_142	cauuuaguagaucaaguguuc	novel_miR_166	uguagggaguagaaugcagc	novel_miR_262	uugugacuagaauuuugcuuc
novel_miR_69	cuuucuaagagaugguacgaacuu	novel_miR_206	uuggugacuacgauuuugauugu	novel_miR_13	cuuucugcuaaagucaucugc
novel_miR_260	aaguggcugccaugugaagugggc	novel_miR_3	agacuuguauagcuuagagcuaga	novel_miR_8	aauugggcugcgaacugguuucgc
novel_miR_192	uaauuacccuuuuuaacugua	novel_miR_257	uuggcccuucucccucucaug	novel_miR_204	uuggauugcugccuuaugagc
novel_miR_111	ucgcaggagagaugacgccgu	novel_miR_70	cuucaaagggcuauuuugaacaau	novel_miR_6	gugauuauaggaaguguuucu
novel_miR_219	cgaacuuugacacauugccaa	novel_miR_123	uugguagugauuauaggaagu	novel_miR_14	agcucuguuggacucucuuug
novel_miR_15	uugccgacuccacccauaccua	novel_miR_162	uuuagcugaauagaaaaaauc	novel_miR_39	auugcgacuuuaguuaaaaaauga
novel_miR_93	uguuugccgggagugcucucc	novel_miR_254	uuuagcugaauagaaaaaauc	novel_miR_100	auuaauuguucccaccgcucu
novel_miR_10	aagagacgaguucccuuuggaauu	novel_miR_235	ucaggucugucauauggauuuagc	novel_miR_232	ucaggucugucauauggauuuagc
novel_miR_85	augugaccguuggagccuagcucc	novel_miR_159	ugauuauaggaaguguuucu	novel_miR_132	augacuugaagauaaugauca
novel_miR_221	gaguguacgaacuuucuaagagag	novel_miR_218	uaaaaucacugucaaacggacucu	novel_miR_173	uucgugacucauugaucuucaauc
novel_miR_249	guuagugacuacggcuuugacugu	novel_miR_80	agugcuucggauuagagugagucu	novel_miR_41	aacccugugugacuccuuauuugg
novel_miR_83	agugauuuuagaaaauguuuuu	novel_miR_92	uuaugaccucgacaaacuuug	novel_miR_140	uggggaugcauguugaaccuuu
novel_miR_141	aaguggcugccaugugaagugggc	novel_miR_115	uaguggaucuuggguacuacc	novel_miR_19	uuugagagugauuuuagaaaa
novel_miR_51	uaauucugggggacaucuacc	novel_miR_26	uauaguacucgacaagugauu	novel_miR_79	cguuugguagugauuauagga
novel_miR_148	uugguagugauuauaggaagu	novel_miR_12	agaaacacuuccuagaaucacugc	novel_miR_44	agaaacacuuccuagaaucacugc
novel_miR_205	ugacgguugaggaaauguggauc	novel_miR_65	cuugagagcuccaauggaugg	novel_miR_56	guuggaagccggugggggacc
novel_miR_181	cugggcuuuuguuucuucgug	novel_miR_18	augugaccguuggagccuagcucc	novel_miR_104	acuucucuuaacccuguagacgug
novel_miR_47	uauuguugaauguagaaauuuaca	novel_miR_175	uaaacacuuucuagaaucacu	novel_miR_4	cgacgggugcgugacgcc
novel_miR_88	guuggaagccggugggggacc	novel_miR_71	agugauuuuaggaaguguuuaua	novel_miR_227	auauuggaugauguuaacaug
novel_miR_217	cagcuuccgguuguucucccc	novel_miR_37	ucggcuccuggaggaugugg	novel_miR_112	ugacaacugaggcuucauuuuuga
novel_miR_86	guggcucaaauguuuuuucucagg	novel_miR_135	aggcgugacacuagugggaacucu	novel_miR_58	ugcauuugcaccugcaccuua

novel_miR_195	gugccugugaggaaccuucac	novel_miR_188	cguuugguagugauuauagga	novel_miR_199	auguauuugagggaaagcaaa
novel_miR_74	cggaaaaucaauaaugagu	novel_miR_78	auuuggcaucucuauaaaugauag	novel_miR_184	uuuuuaggacuguaccuuagguac
novel_miR_174	uuagaugaucaucaacaaaca	novel_miR_201	uaaugcgugaugacuuaaaaugac	novel_miR_107	uuugagagugauuuuagaaaa
novel_miR_116	aggauguacaagacuaguauuugu	novel_miR_124	ucuuaccaacaccucccauucc	novel_miR_203	uccuaaaaucacugucaaacg
novel_miR_24	uggugaaccaaauaacucugg	novel_miR_237	guggguaucuucucuacauga	novel_miR_258	ugagacucgguggcuugaacaacc
novel_miR_50	auauuggaugauguuaacaug	novel_miR_164	cugaacucucucccucauggcc	novel_miR_193	augaucuaauaaugaacc
novel_miR_156	cauuuaguagaucaaguguuc	novel_miR_61	uugguagugauuauaggaagu	novel_miR_82	ugaccggcucuuaucucucaug
novel_miR_264	ggagaugauuuuauagacgug	novel_miR_5	ucuuaccaacaccucccauucc	novel_miR_165	uggggaugcauguugaaccuuu
novel_miR_168	uugugacuagaauuuugcuuc	novel_miR_45	uggggaugcauguugaaccuuu	novel_miR_157	gaagaagaaccugaagag
novel_miR_46	acaagacuuaggaagaaugcaccu	novel_miR_53	ccuuccggcaucuggacaucc	novel_miR_139	cucgacucugauaccacuuguagg
novel_miR_59	cagacggauccacaugauaga	novel_miR_213	uuucuguuuucuauucuuaagaac	novel_miR_239	acuaagacuucauuuuuaaacuga
novel_miR_48	gaugaggaucuuuggaugcaa	novel_miR_1	uuccggcaucuggacaucccc	novel_miR_191	guuagugacuacggcuuugacugu
novel_miR_136	aagaaaaacaggucugugaaaaac	novel_miR_9	auuaauuguucccaccgcucu	novel_miR_186	uaaacacuuucuagaaucacu
novel_miR_131	uaguuggcuuucgauuuguacacc	novel_miR_94	agaaacacuuccuagaaucacugc	novel_miR_32	acugaacucgucuauugaaaagau
novel_miR_40	aagaaucuuuuguacaucuguauc	novel_miR_211	auugagacuucauuuuuuaacuga	novel_miR_152	ugaggcucauacgaucuggagaug
novel_miR_75	aaacacuuucuagaaucacu	novel_miR_2	uuggauucgcgcacaaacucg	novel_miR_150	cgagauguauggauuggaucc
novel_miR_155	agugaucuuaggaaauauuuuuaa	novel_miR_43	agugaucuuaggaaauauuuuuaa	novel_miR_81	acaguguauuucucugaaagaagg
novel_miR_223	ccaauggauacagaucuugaaagu	novel_miR_11	uaaaaucacugucaaacggacucu	novel_miR_228	augaucuaauaaugaacc
novel_miR_25	agugauuuuaggaaguguuuaua	novel_miR_251	uugguagugauuauaggaagu	novel_miR_194	cucuucugaugaauaaaugga
novel_miR_229	cuuaacccuguagacguguuuuaa	novel_miR_102	aucggaagugugccaagaucaagg	novel_miR_63	gggcaauuacuccauuggcagu

Table S5. Sequences of miRNAs for qRT-PCR

Gene ID	Primes	Sequences(5'→3′)
vvi-miR828a	FORWARD	CCTGCTGGTCTTGCTCAAATGAG
	REVERSE	ATCCAGTGCAGGGTCCGAGG

vvi-miR156i	FORWARD	CGCGCGTTGACAGAAGATAGA
	REVERSE	ATCCAGTGCAGGGTCCGAGG
vvi-miR398b	FORWARD	CGCTTGCCTGTGTTCTCAGGT
	REVERSE	ATCCAGTGCAGGGTCCGAGG
vvi-miR171c	FORWARD	AACCGGTGATTGAGCCGTGC
	REVERSE	ATCCAGTGCAGGGTCCGAGG
vvi-miR171e	FORWARD	AACTTGATTGATTGAGCCGCGC
	REVERSE	ATCCAGTGCAGGGTCCGAGG
vvi-miR3630-5p	FORWARD	CCACGGAGTGCAAGTGACGATA
	REVERSE	ATCCAGTGCAGGGTCCGAGG
vvi-miR398a	FORWARD	CGCTTGCCTGTGTTCTCAGGTCA
	REVERSE	ATCCAGTGCAGGGTCCGAGG
pCa-vvi-miR171c	FORWARD	AACCGGTGATTGAGCCGTGC
	REVERSE	ATCCAGTGCAGGGTCCGAGG
VvMYB154	FORWARD	ACTGGTGGTAACTGGATTGCTCTTC
	REVERSE	TCCATGCTTGATGTCTGGTCTGAG
U6	FORWARD	CTAATGGGGAGTGGGGAAGTA
	REVERSE	GTCTGGATGGACAATGTTGAT

Figure S1. Differential miRNA heatmap (CS/CS vs CS/140R)

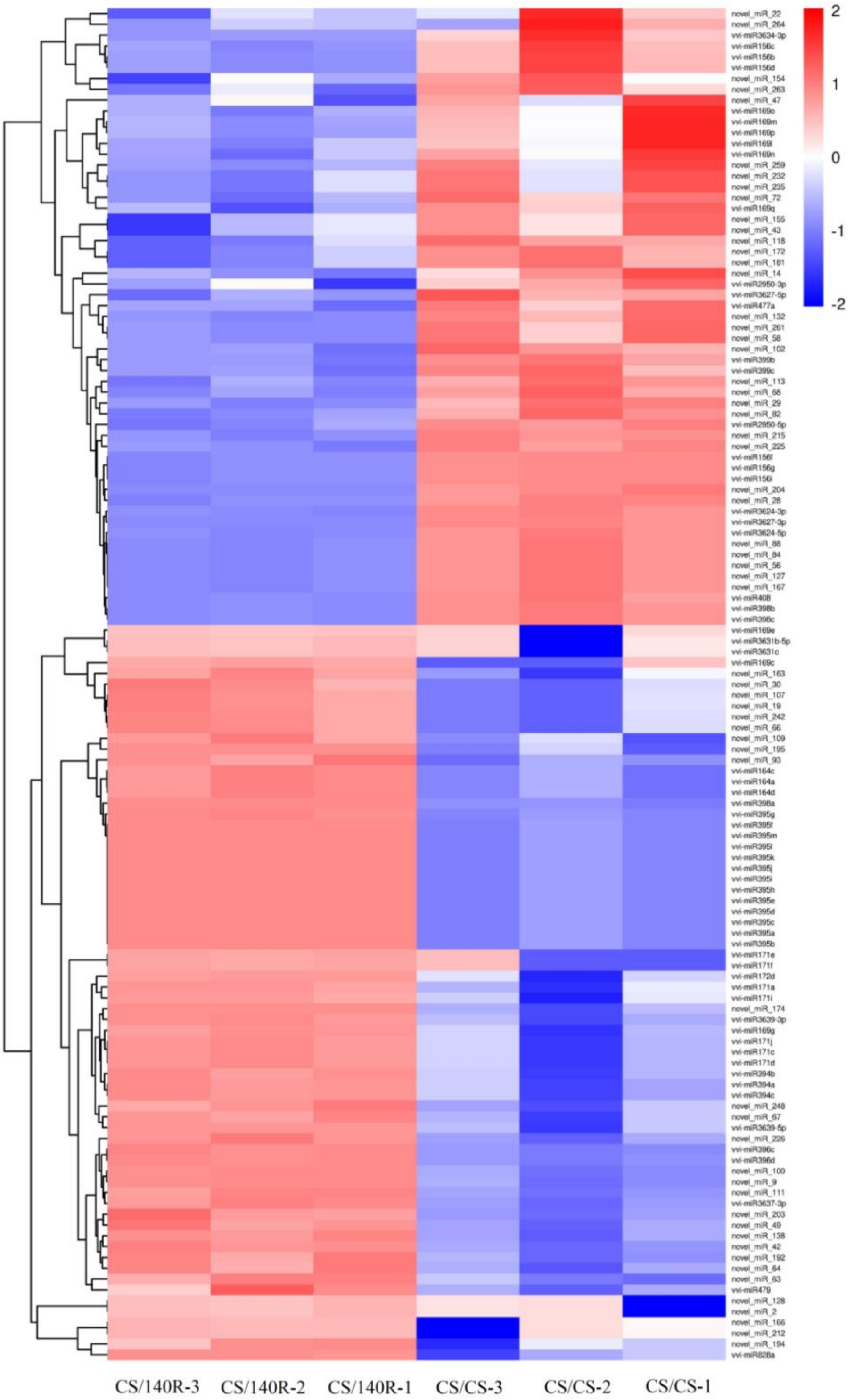

Figure S2. Differential miRNA heatmap (CS /CS vs 140R/140R)

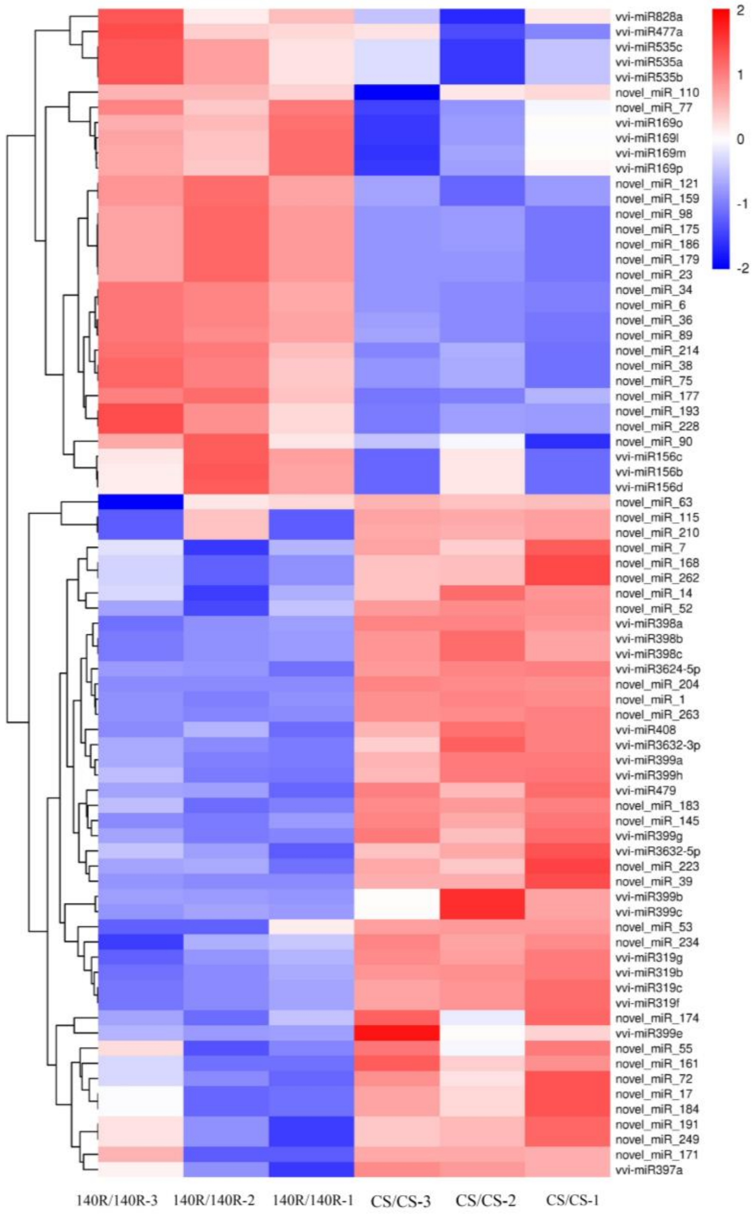

Figure S3. Annotation of miRNA target genes : NR homologous species distribution

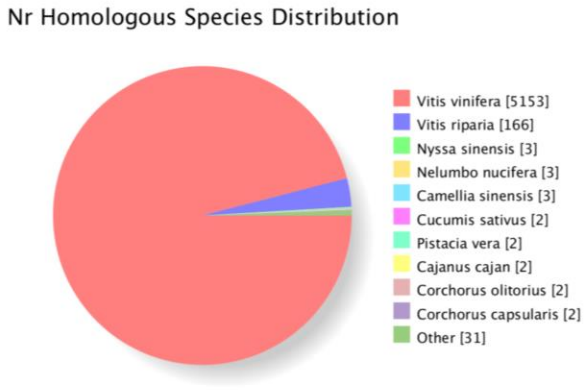

Figure S4. Annotation of miRNA target genes. (A) COG classification statistics (C) KOG classification statistics (B) differential miRNA KEGG analysis (E) differential miRNA GO analysis

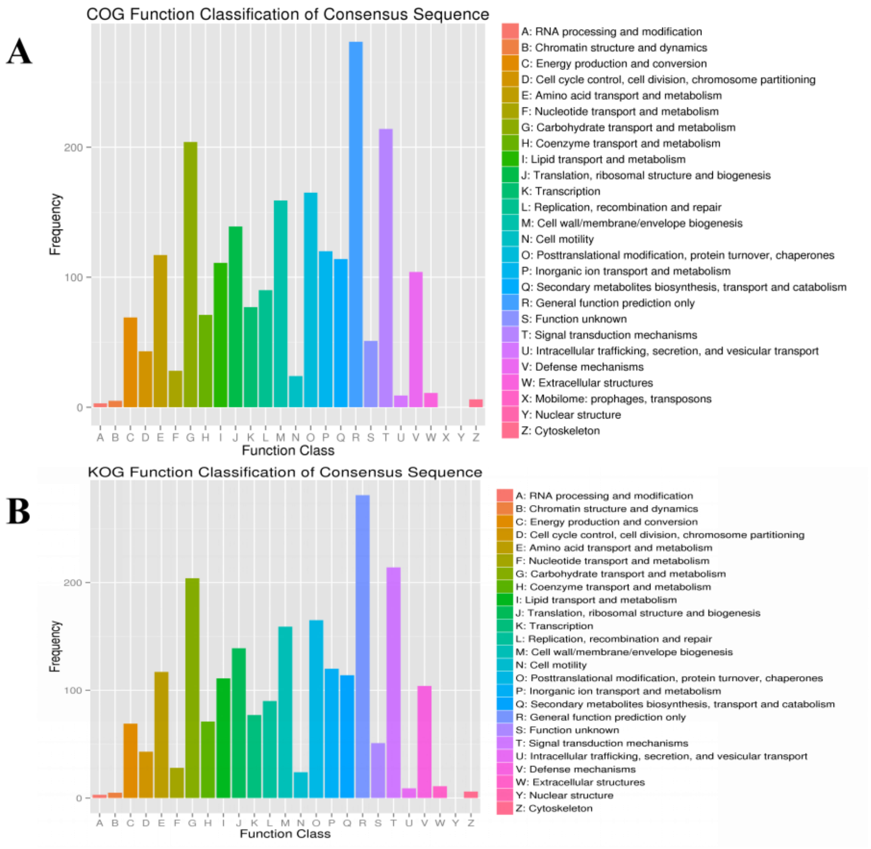

Supplement: Supplementary file 1 [file plants-13-03057-s001.zip › plants-3230907-supplementary material.pdf]
